# Effect of postoperative systemic therapy on pulmonary adenocarcinoma with unexpected pleural spread detected during thoracotomy or thoracoscopy

**DOI:** 10.18632/oncotarget.23686

**Published:** 2017-12-26

**Authors:** Chi-Lu Chiang, Lei-Chi Wang, Hsiang-Ling Ho, Chun-Ming Tsai, Yi-Chen Yeh, Wen-Hu Hsu, Teh-Ying Chou, Chao-Hua Chiu, Yu-Chung Wu

**Affiliations:** ^1^ Division of Thoracic Oncology, Department of Chest Medicine, Taipei Veterans General Hospital, Taipei, Taiwan; ^2^ Faculty of Medicine, School of Medicine, National Yang-Ming University, Taipei, Taiwan; ^3^ Department of Pathology and Laboratory Medicine, Taipei Veterans General Hospital, Taipei, Taiwan; ^4^ Department of Oncology, Taipei Veterans General Hospital, Taipei, Taiwan; ^5^ Institute of Clinical Medicine, School of Medicine, National Yang-Ming University, Taipei, Taiwan; ^6^ Division of Thoracic Surgery, Department of Surgery, Taipei Veterans General Hospital, Taipei, Taiwan

**Keywords:** pulmonary adenocarcinoma, pleural spread, epidermal growth factor receptor, tyrosine kinase inhibitor, postoperative therapy

## Abstract

**Background:**

Occasionally, malignant pleural disease is only detected unexpectedly during surgery in patients with pulmonary adenocarcinoma. Previous studies mostly focused on the role of main tumor resection on patient's outcome, barely addressing the position of postoperative systemic therapy.

**Methods:**

The medical records of 5321 non-small cell lung cancer patients who underwent thoracic surgery between January 1990 and December 2012 were reviewed. Pulmonary adenocarcinoma patients with unexpected pleural spread noted during surgery were included. The clinical and postoperative treatment variables were assessed for correlation with overall survival.

**Results:**

In 134 patients identified, main tumor resection was performed in 87 (64.9%) patients, while 89 (66.4%) and 57 (42.5%) patients received postoperative chemotherapy and epidermal growth factor receptor- tyrosine kinase inhibitor (EGFR -TKI) therapy, respectively. Overall, the 5-year survival rate was 30.2% and median survival time was 29.3 months. Multivariate analysis showed main tumor resection and EGFR-TKI therapy were associated with better survival. Mutational status of EGFR was available in 57 patients and 43 (75.4%) had activating mutations. Resection of the main tumor conferred a better outcome in patients without EGFR mutation or with unknown EGFR mutation status and had not been treated with EGFR-TKI therapy (*P* = 0.003), but not in those with activating EGFR mutation and had been treated with EGFR-TKI (*P* = 0.857).

**Conclusions:**

In pulmonary adenocarcinoma patients with unexpected pleural spread detected during surgery, main tumor resection and EGFR-TKI therapy correlated with better survival. Identifying EGFR mutation status before surgery can provide useful information for clinical decision during surgery.

## INTRODUCTION

The discovery of epidermal growth factor receptor (EGFR) mutations has resulted in a paradigm shift in the management of advanced NSCLC [1]. Numerous studies showed that in patients with advanced NSCLC carrying EGFR mutations, treatment with epidermal growth factor receptor –tyrosine kinase inhibitors (EGFR-TKIs) improved chances of survival [2–6]. Most patients with EGFR sensitizing mutations and who respond to EGFR-TKIs present with adenocarcinoma [7]. While adenocarcinoma usually presents at the lung periphery and is frequently associated with an initial malignant pleural effusion, a positive association between EGFR mutation and the presence of malignant pleural effusion has been reported [8–10].

Patients with malignant pleural disease including malignant pleural effusion and/or malignant pleural nodules are classified as stage IV (M1a) in the current staging system due to poor prognosis [11, 12]. Thus, lung adenocarcinoma patients with malignant pleural disease are considered inoperable, and are candidates for systemic therapy. However, the clinical presentation of pleural carcinomatosis has a wide range of manifestations; varying from pleural effusion large amount enough to be diagnosed by plain radiography to minimal disease which is only detected incidentally during a thoracotomy. Performing resection of the main tumor for patients with unexpected pleural spread detected during surgery is a matter of debate [13–17]. One of the limitations of previous studies was that the various confounding postoperative treatments were not taking into account. Since there have been remarkable changes in systemic therapy for advanced pulmonary adenocarcinoma, resulting in significant improvement in survival outcomes [18, 19]. In this study, we decided to further persuade the prognostic factors, particularly postoperative systemic therapy (POST), in pulmonary adenocarcinoma patients who had unexpected pleural spread detected during thoracic surgery.

## RESULTS

### Patient characteristics

**Figure 1:**
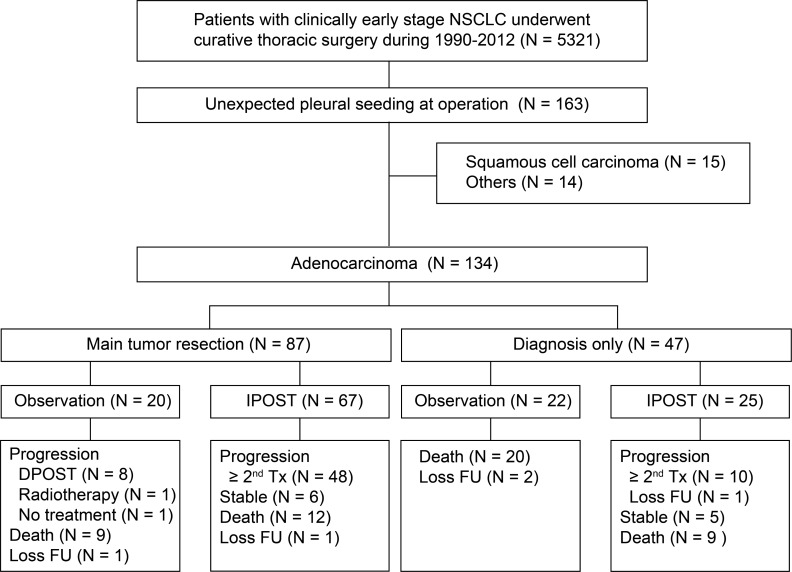
Diagram describing recruitment strategy of study participants and follow-up (IPOST: immediate postoperative systemic therapy; DPOST: delayed postoperative systemic therapy; Tx: treatment).

Between January 1990 and December 2012, 5321 patients with a clinical diagnosis of lung cancer underwent thoracic surgery in our institute. A total of 163 (3.1%) patients, mostly adenocarcinomas (*n* = 134, 82.2%) had unexpected pleural spread detected during surgery (Figure 1). In patients with pulmonary adenocarcinoma, the median age was 63.3, and 55.2% of the patients were man (Table 1). Most surgeries were thoracotomies (73.9%), although video-assisted thoracoscopic surgery was the predominant surgical procedure after 2008. Intraoperative pleural effusion cytology examination was performed in 29 patients (30.2%). Main tumor resection was performed in 87 patients (64.9%); 58 patients had lobectomy and 29 patients underwent wedge resection. There was no significant difference between diagnosis by thoracotomy or by thoracotomy and the selection of following surgical procedures (as shown in Supplementary Table 1). Ninety-two (68.7%) patients received immediate POST and the median time to treatment was 1.1 months (range: 0.2–2.9 months).

**Table 1:** Demographic and clinicopathological characteristics of patients

Variables	Number (%)
Total	134 (100)
Age, y (median)	63.3 (32.4–81.1)
Sex	
Male	74 (55.2)
Female	60 (44.8)
Smoking	
Never smoker	96 (71.6)
Ever/Current smoker	38 (28.4)
Tumor location	
Left	67 (50)
Right	67 (50)
Tumor size	
≤3 cm	72 (53.7)
>3 cm	56 (41.8)
Pathological N stage	
N0	31 (23.1)
N1	19 (14.2)
N2	38 (28.4)
Not examined	46 (34.3)
Malignant pleural disease^*^	
MPE only	3 (3.1)
MPN only	69 (71.9)
MPN + MPE	24 (25.0)
Surgical approach method	
Thoracotomy	99 (73.9)
VATS convert to thoracotomy	10 (7.4)
VATS	25 (18.7)
Surgical procedure for main tumor	
Lobectomy (standard resection)	58 (43.3)
Wedge resection (limited resection)	29 (21.6)
No main tumor resection	47 (35.1)
CEA	
Normal	47 (35.1)
High	45 (33.6)
Unknown	42 (31.3)

^*^Data available in 96 patients.

Abbreviations: MPE: malignant pleural effusion, MPN: malignant pleural nodule, VATS: video-assisted thoracoscopic surgery, CEA: carcinoembryonic antigen.

### Postoperative systemic therapy

The details of the immediate POST are summarized in Table 2. Overall, 89 (66.4%) patients had received chemotherapy, out of which 73 patients were treated immediately after surgery. Fifty-seven (42.5%) patients had received EGFR-TKI therapy, out of which 19 were treated immediately after surgery. EGFR mutation testing was performed for all patients with EGFR-TKI treatment history and activating mutation were found in 43 patients (exon 19 deletion in 24, L858R in 17, and G719C and exon 19 deletion + L858R in one each).

**Table 2:** Immediate postoperative systemic therapy according to the EGFR mutation status

Group	Unknown *N* = 77	Wild type *N* = 14	Mutant *N* = 43	All *N* = 134
Main tumor resection	45	9	33	87
Observation only	12	0	0	12
Immediate systemic therapy	27	7	33	67
Chemotherapy	27	7	24	58
EGFR-TKI	0	0	9	9
Systemic therapy after recurrence	6	2	0	8
Chemotherapy	6	0	0	6
EGFR-TKI	0	2	0	2
Diagnostic thoracotomy or thoracoscopy	32	5	10	47
Observation only	22	0	0	22
Immediate systemic therapy	10	5	10	25
Chemotherapy	10	3	2	15
EGFR-TKI	0	2	8	10

### Overall survival analysis

**Figure 2:**
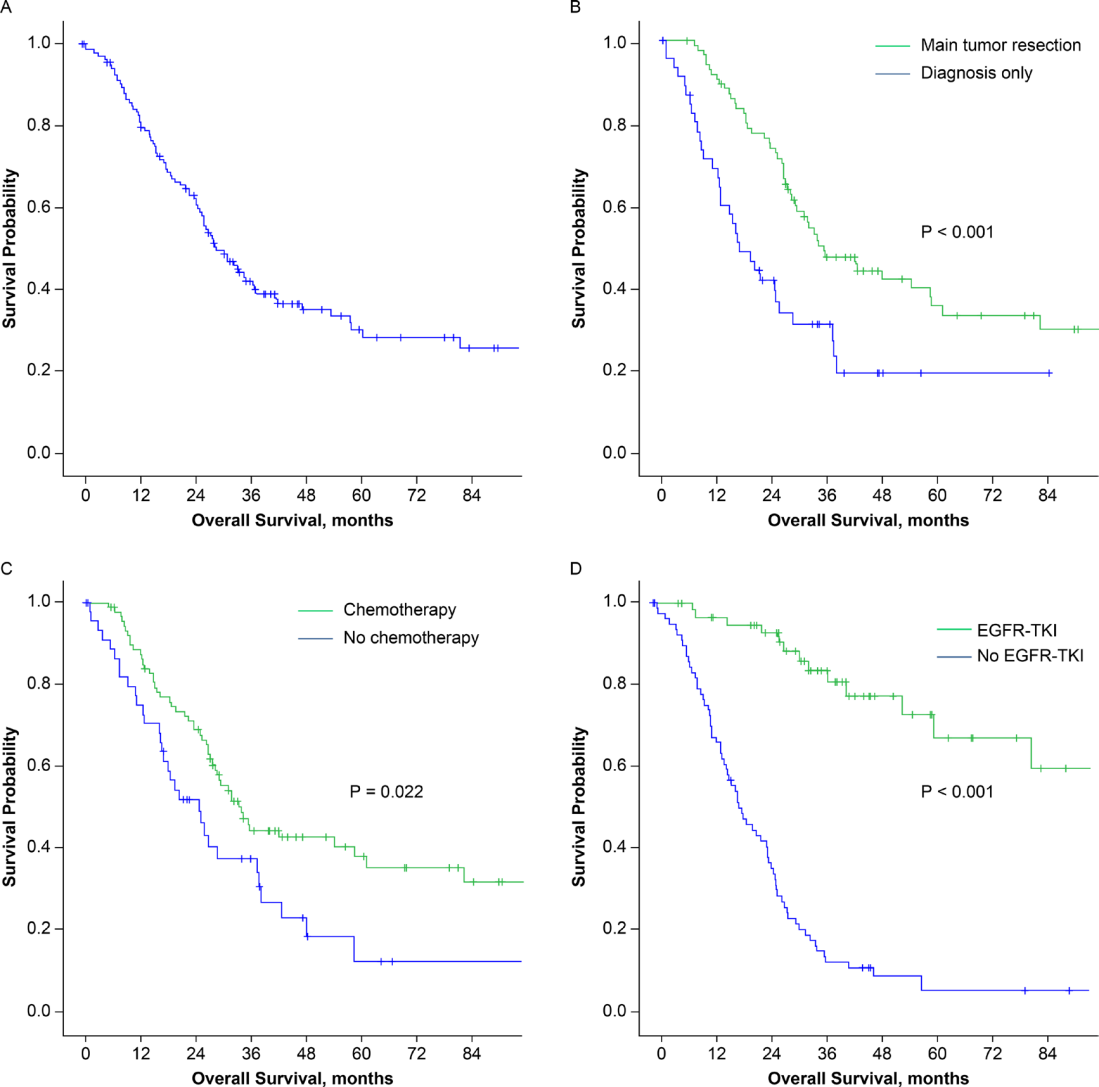
Kaplan-Meier survival curves for (**A**) all 134 patients, (**B**) patients who received (green) or did not receive (blue) main tumor resection, (**C**) patients who received (green) or did not receive (blue) postoperative chemotherapy, (**D**) patients who received (green) or did not receive (blue) postoperative EGFR-TKI treatment. (EGFR-TKI: epidermal growth factor receptor –tyrosine kinase inhibitor).

Overall, the 5-year survival rate was 30.2% and the median survival time was 29.3 months (95% CI, 23.4–35.2 months; Figure 2A). Patients who received main tumor resection had longer survival than patients who did not (median 35.3 versus 17.0 months, *P* < 0.001; Figure 2B). Significant survival differences were also observed between patients who underwent postoperative chemotherapy and those who did not (median, 33.8 versus 24.7 months, *P* = 0.022; Figure 2C) and between patients who underwent postoperative EGFR-TKI treatment and those who did not (median, 111.1 versus 18.6 months, *P* < 0.001; Figure 2D). The timing of the POST and tumor size were also revealed to be prognostic factors in univariate analysis. However, in multivariate analysis, only main tumor resection and EGFR-TKI therapy correlated with better survival (*P* = 0.013 and < 0.001, respectively, Table 3).

**Table 3:** Univariate and multivariate analyses of prognostic factors by using Cox proportional hazards model

Variables	*N* (%)	Univariate analysis		Multivariate analysis	
		Hazard Ratio (95% CI)	*P* value	Hazard Ratio (95% CI)	*P* value
Age			0.183		
<65	74 (55.2)	1			
≥65	60 (44.8)	1.333 (0.873–2.037)			
Sex			0.306		
Male	74 (55.2)	1			
Female	60 (44.8)	0.798 (0.518–1.229)			
Smoking			0.964		
Never	96 (71.6)	1			
Ever	38 (28.4)	1.011 (0.637–1.603)			
CEA level			0.288		
Normal	47 (35.1)	1			
High	45 (33.6)	1.373 (0.765–2.465)			
Tumor size			0.002		0.278
≤3 cm	72 (53.7)	1		1	
>3 cm	56 (41.8)	1.968 (1.269–3.052)		1.29 (0.815–2.042)	
Pathological N2			0.194		
No	96 (71.6)	1			
Yes	38 (28.4)	1.365 (0.854–2.184)			
Main tumor resection			<0.001		0.013
No	47 (35.1)	1		1	
Yes	87 (64.9)	0.45 (0.288–0.704)		0.484 (0.273–0.859)	
Immediate postoperative therapy			<0.001		0.200
No	42 (31.3)	1		1	
Yes	92 (68.7)	0.382 (0.249–0.587)		0.604 (0.279–1.307)	
Postoperative Chemotherapy			0.024		0.190
No	45 (33.6)	1		1	
Yes	89 (66.4)	0.602 (0.388–0.935)		1.737 (0.760–3.971)	
Postoperative EGFR-TKI use			<0.001		<0.001
No	77 (57.5)	1		1	
Yes	57 (42.5)	0.15 (0.087–0.257)		0.179 (0.098–0.328)	

### Interaction between main tumor resection and EGFR-TKI treatment

**Figure 3:**
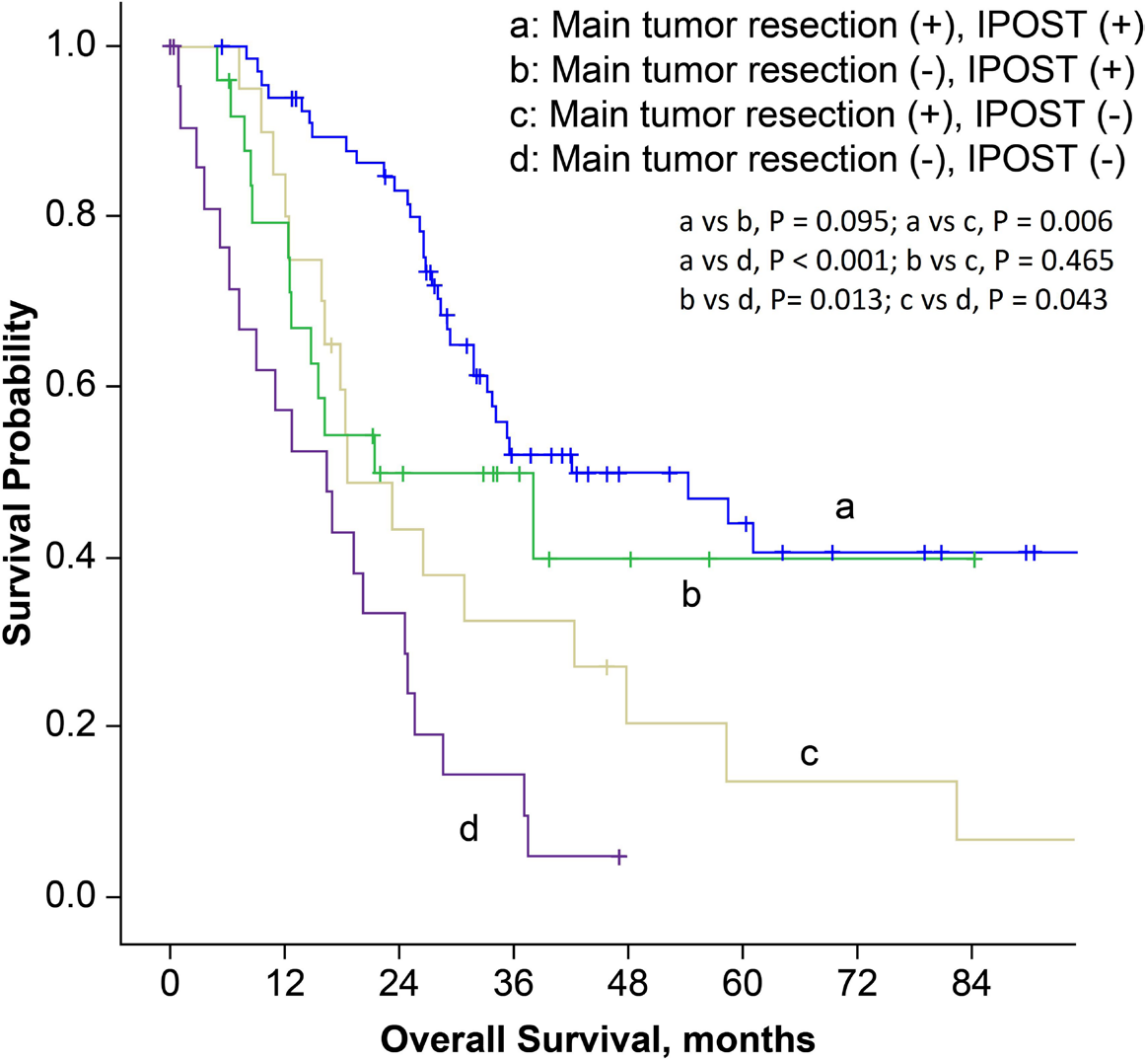
Kaplan-Meier survival curves for patients stratified by surgical procedures and postoperative systemic therapy (IPOST: immediate postoperative systemic therapy).

**Figure 4:**
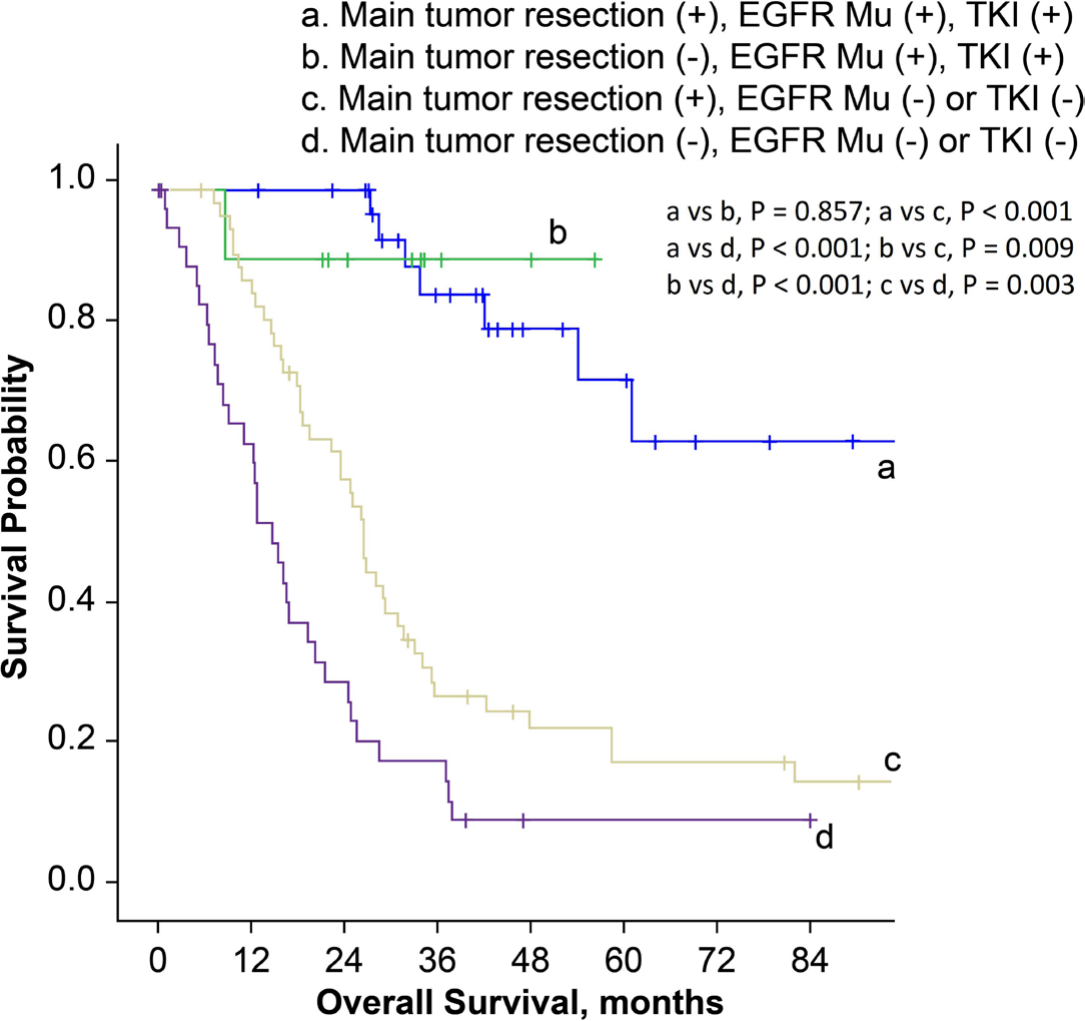
Kaplan-Meier survival curves for patients stratified by surgical procedures, EGFR mutation status and history of EGFR-TKI treatment (EGFR Mu: epidermal growth factor receptor mutation; TKI: tyrosine kinase inhibitor).

In order to establish prognostic correlation, we stratified patients according to surgical procedure and postoperative management. The median overall survival time for patients who had received main tumor resection and immediate POST was 42.1 months (95% CI, 15.3–68.9 months), which was much better compared to 18.6 months (95% CI, 10.9–26.3 months) in patients who had received main tumor resection but not immediate POST, and 16.5 months (95% CI, 7.6–25.4 months) in patients with diagnostic thoracic surgery and no instantaneous subsequent therapy (*P* = 0.006 and < 0.001, respectively; Figure 3). Then we stratified patients according to the surgical procedure, EGFR mutation status and history of EGFR-TKI treatment. Main tumor resection was not a prognostic factor in patients with EGFR mutations and EGFR-TKI treatment history (*P* = 0.857). However, in patients with EGFR wild-type, or in patients with unknown EGFR status and without EGFR-TKI treatment history, the overall survival was better for patients who received main tumor resection (*P* = 0.003, Figure 4).

## DISCUSSION

In this study, when we incorporated the detailed history of postoperative therapy into our analysis, we found that the conclusion of our previous study in patients with unexpected spread at thoracotomy had to be modified according to the patients’ EGFR mutation status. Main tumor resection was still a valuable procedure for patients who were negative or unknown for EGFR mutation. However, for patients who had sensitizing EGFR mutations and had been treated by EGFR-TKI, we did not observe any survival benefit conferred by main tumor resection.

In the present study, the 5-year survival rate was 30.2% and median survival time was 29.3 months. In another study, using the Japanese lung cancer registry in 2004, Iida, T. *et al.* reported a very similar outcome in 313 surgical patients who had pleural carcinomatosis first detected during thoracotomy: a 5-year survival rate of 29.3% and a median survival time was of 34.0 months [20]. The result of this subgroup of stage IV disease was comparable to that in patients with stage IIB to IIIA disease [21]. Taken together with our findings, this data suggest that patients with minimal malignant pleural disease have a relatively favorable prognosis which is much different from patients, with clinically overt malignant pleural disease. Distinctive treatment strategy is thus indicated based on the nature of the disease.

In this study, main tumor resection was performed in about two-thirds of patients (64.9%), and most patients received standard surgery (lobectomy with radical lymph node dissection). We found that main tumor resection could confer survival benefit in lung adenocarcinoma patients with unexpected pleural spread detected at thoracotomy or thoracoscopy, which was in agreement with our previous results [15, 17, 20, 22]. Moreover, we found that POST also contributed to survival advantage, an aspect that has never been addressed before. In the present study, 100 patients (74.6%) received POST and 92 of them were treated immediately after surgery. In our study, patients who received POST, either as chemotherapy or as EGFR-TKI therapy had longer overall survival than those who did not. The 5-year survival rate was 43.9% in patients who received main tumor resection and immediate POST (Figure 3); this finding highlights the importance of systemic therapy after surgery in this group of patients.

Undoubtedly, with the advent of targeted therapy, the remarkable efficacy of EGFR-TKI in patients with sensitizing EGFR mutations further strengthens the value of POST in patients with unexpected pleural spread. Indeed, in patients with EGFR mutations, the effect of postoperative EGFR-TKI treatment seemed to overwhelm the influence of main tumor resection on overall survival. In agreement with other published studies, we demonstrated that main tumor resection could confer a survival benefit in patients with negative or unknown EGFR mutation status. Surprisingly, we saw no evidence to support the value of main tumor resection in extending survival outcome in patients who had sensitizing EGFR mutations with EGFR-TKI POST history. To the best of our knowledge, this is the first study to discuss the role of EGFR mutations and EGFR-TKI treatment in pulmonary adenocarcinoma patients with unexpected pleural spread detected during thoracotomy or thoracoscopy.

According to the current guideline, EGFR mutation testing is indicated at the time of diagnosis for patients with advanced NSCLC [23, 24]. Whether EGFR mutation testing should be done in clinical stage I, II or III disease remains controversial. We demonstrate here the differential survival outcomes of main tumor resection in patients with and without EGFR mutation. Our study results suggest that EGFR mutation status may assist surgeons in surgical decision making when pleural spread is found unexpectedly during thoracotomy. In addition, our results also imply that in patients with EGFR sensitizing mutations, surgery may not provide additional value over EGFR-TKI therapy, and can be avoided. This could potentially reduce surgical risks, avoid unnecessary tissue damage, and shorten time of recovery. On the other hand, we believe that in patients with negative or unknown EGFR mutation status, main tumor resection is beneficial and recommended if feasible. Thus, we advocate that EGFR mutation testing should be done at the time of diagnosis in not only patients with advanced disease but also in those with early stage disease who plan to undergo thoracic surgery. Interestingly, in other solid tumors, molecular testing is routinely performed regardless of disease stage, such as HER2 testing in invasive breast cancer [25].

This retrospective study has several limitations. First, there was inevitable surgeon bias in selecting the operative procedures for patients with unexpected pleural spread. Surgeons tend to perform diagnostic thoracotomy without main tumor resection in patients with extensive pleural involvement or poor physical fitness. Both are potential prognostic factors but are difficult to measure objectively. Therefore, it was not possible to incorporate these parameters into the regression model we used for analysis. Second, EGFR mutation testing was only performed in patients who had received EGFR-TKI therapy but not in the rest of the patients. This is related to the fact that the study period was long, and aged archived paraffin blocks are not suitable for molecular testing. Additionally, EGFR mutation per se seems not to be a prognostic factor [26, 27]. Therefore, the incompleteness of the EGFR mutation status will hardly change our study results. Third, we did not check other driver mutations, such as gene rearrangements of ALK and ROS1. However, it has been shown that patients who had driver mutations and had received matched targeted therapy did live longer but patients who had not received targeted therapy had the similar outcome to those who did not have driver mutations [28]. None of our patients had received targeted therapy other than EGFR-TKI and therefore, this missing data will not influence our results. Finally, 22 of our study subjects (16.4 %) of our study subjects did not receive systemic therapy after diagnostic thoracotomy and it may be explained, at least partly, by cultural difference. Previous reports showed a significant percentage (22.75%) of Taiwanese patients refuse treatment after lung cancer diagnosis [29]. Besides, 21 of the 22 patients (95.5%) were diagnosed to have expected pleural spread before 2000, which indicated that cytotoxic chemotherapy is the only systemic treatment at that time. (Gefitinib was reimbursed in Taiwan after Nov. 2004). Patients may refuse systemic treatment due to the concern of side effects.

In conclusion, both main tumor resection and postoperative EGFR-TKI therapy were associated with better survival in pulmonary adenocarcinoma patients with unexpected pleural spread noted during thoracic surgery. Testing for EGFR mutations should be considered in not only advanced but also in early stage lung adenocarcinoma patients. A randomized prospective study is needed to confirm the role of surgery in this subset of patients who have driver mutations detected before surgery.

## METHODS

### Patients

Medical records of clinical early stage lung cancer patients who had undergone thoracic surgery at this tertiary medical center were reviewed. The preoperative work-up included chest radiograph and computed tomography (CT) of chest, cardiopulmonary function evaluation, and a thorough search for distant metastases, including brain CT scan and whole body bone scan. All surgeries were performed with curative intent and the surgeon decided on the extent of surgery. The details regarding POST were obtained from the patients’ medical records. Immediate POST was defined as the systemic therapy delivered within three months post-surgery.

The definition of “unexpected pleural spread” was as follows: (1) preoperative survey indicated a resectable lung tumor and the patient was scheduled for a standard lobectomy, (2) pleural disease was first detected during surgery, (3) pleural metastasis was proved pathologically. The operative finding, the method of pulmonary resection, and the patient characteristics, like age, sex, tumor size, smoking history, and preoperative carcinoembryonic antigen level were reviewed. This study was conducted in accordance with the Declaration of Helsinki. The Institutional Review Board of Taipei Veterans General Hospital approved this study and informed consent was waived (IRB No. 2014-04-003CC).

### Tumor samples and EGFR mutation testing

Tumors from patients who underwent EGFR-TKI treatment were evaluated for EGFR mutation status. Formalin-fixed, paraffin-embedded tissue was the main sample type that was used. Briefly, one of the consecutive sections was stained with hematoxylin and eosin and reviewed by pathologists to select a region which was enriched by tumor cells. The selected region was marked on a deparaffinized tissue section, and manually microdissected. Genomic DNA was extracted by PicoPure DNA extraction kit (Arcturus/Applied Biosystems, Foster City, CA, USA), and quantified by using Nanodrop 2000 (Thermo Fisher Scientific, Waltham, MA, USA).

Two EGFR mutation testing methods were used in the study period. From 2008 to 2011, we used direct sequencing to detect mutations at exons 18 to 21. The sequence variations were confirmed by multiple independent polymerase chain reaction amplifications and repeated sequencing reactions as previously described [30]. After 2011, the majority of specimens were tested using the mutant allele-specific, real-time PCR-based, mutation detection technology [31].

### Statistical analyses

Data are presented as number (percentage) or median (range) unless otherwise stated. Overall survival was defined as the interval between the date of surgery and the date of death or last follow-up (cutoff date, August 31, 2014). Survival curves were plotted by the Kaplan-Meier method and compared by the log-rank test. Multivariate Cox proportional hazard model was used to identify prognostic factors. All factors with *P* < 0.1 in univariate analyses were included in the Cox regression analysis using enter method. A significant difference was defined as a *P* < 0.05. When multiple comparisons were performed, the cutoff level of α error was reduced using Bonferroni correction. Analyses and graphing were performed using SPSS statistics software (SPSS Inc., Chicago, IL, USA).

## SUPPLEMENTARY TABLE



## Abbreviations

CT: computed tomography
EGFR: epidermal growth factor receptor
EGFR-TKI: epidermal growth factor receptor –tyrosine kinase inhibitor
NSCLC: non-small cell lung cancer
POST: postoperative systemic therapy
CI: confidence interval
