# Supplementary material for: Effect of postoperative systemic therapy on pulmonary adenocarcinoma with unexpected pleural spread detected during thoracotomy or thoracoscopy
Source: Oncotarget. 2017 Dec 26;9(4):5435–44. doi: 10.18632/oncotarget.23686 (PMC5797062; doi:10.18632/oncotarget.23686)
Supplement: Supplementary file 1 [file oncotarget-09-5435-s001.pdf]

Effect of postoperative systemic therapy on pulmonary adenocarcinoma with unexpected pleural spread detected during thoracotomy or thoracoscopy

SUPPLEMENTARY MATERIALS

Supplementary Table 1: All study patients stratified by surgical approach method and surgical procedure

	Main tumor resection (lobectomy or Wedge resection)	Diagnosis only	*P* value
Thoracotomy	64	35	0.909
VATS	23	12	

VATS: video-assisted thoracoscopic surgery.
